# Breaking down to rebuild: lymphatic ablation enhances osteoclast-driven regeneration

**DOI:** 10.1172/JCI201199

**Published:** 2026-02-02

**Authors:** Matthijs Luxen, Francesca Lazzeri-Barcelo, Ralf H. Adams

**Affiliations:** Department of Tissue Morphogenesis, Max Planck Institute for Molecular Biomedicine, Münster, Germany.

## Abstract

The fingertip is one of the only known complex structures in mammals that can fully regenerate following amputation. This phenomenon can be studied in mice using the amputation of the digit tip, the regenerative success of which has been shown to be reliant on effective bone clearance prior to new bone formation. In this issue of the *JCI*, Vishlaghi et al. investigated whether local lymphatic vessels are involved in this process. Interestingly, they found that inhibiting lymphangiogenesis resulted in accelerated clearance of damaged tissue and bone, thereby improving subsequent digit regeneration. This study is the first to our knowledge to report lymphatic involvement in digit regeneration and raises questions regarding the underlying mechanisms at play.

## Mouse digit tip amputation models complex tissue regeneration

Regeneration of complex body parts composed of highly diverse cell types and tissue structures is rare in mammals. However, the fingertip — formed of skin, nail, bone, connective tissues, nerves, and vasculature — is a unique case. Both humans and mice can naturally regenerate digit tips, and mouse digit tip regeneration is a long-standing model used to study complex mammalian tissue regeneration (1) with the goal of translating findings to therapeutic interventions in humans.

**Figure 1:**
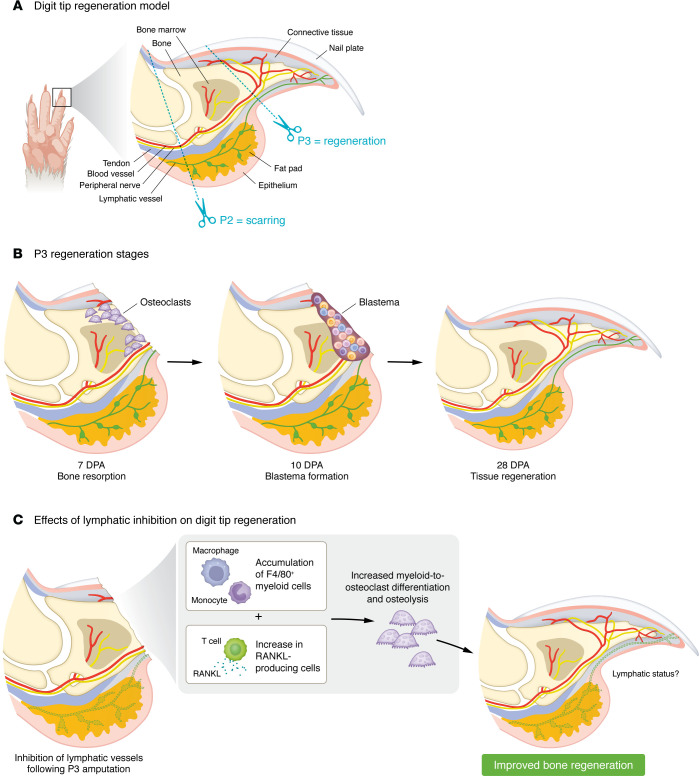
Lymphatic ablation promotes regeneration in a P3 digit amputation model. (**A**) Overview of tissue structure within the digit tip. Amputation along the P2 axis leads to scarring and fibrosis without regeneration, whereas amputation along the more distal P3 axis leads to full regeneration. (**B**) Following P3 digit tip amputation, the damage site undergoes an osteoclast-driven bone resorption phase (7 days post-amputation [DPA]), followed by the formation of the regenerative blastema (10 DPA). At 28 DPA, the digit tip is fully regenerated. (**C**) Vishlaghi et al. (9) showed that inhibition of lymphatics during P3 digit amputation led to accumulation of both F4/80^+^ myeloid cells and RANKL-producing T cells at the damage site. RANKL promoted myeloid-to-osteoclast differentiation, thereby contributing to increased osteolytic activity. More efficient clearance of the amputated bone stump allowed for faster and improved bone regeneration.

The digit tip consists of the distal phalangeal bone (P3), flanked by connective tissue and a ventral fat pad, with blood vessels and nerves running along the lateral sides. Epithelium encases the digit and the nail extends along the top (Figure 1A). P3 amputation, used in most regeneration studies, removes the nail and distal bone tip while sparing the bone marrow and fat pad. Amputated P3 digit tips are fully regenerated in under a month in adult mice, restoring structure and function. In contrast, a P2-level amputation, which removes more bone and disrupts the bone marrow cavity, results in scarring and failed regeneration (1), indicating important limits to the regenerative process and serving as a useful comparison for testing interventions.

After amputation, regeneration begins with an initial inflammatory and wound closure phase, which is followed by osteolysis, consisting of the removal of the distal bone stump by osteoclasts (2) (Figure 1B). Osteolysis is crucial for successful regeneration, as increased osteoclast activity correlates with improved outcomes (2–4). The next critical step is the formation of a blastema, a mass of proliferating progenitor cells derived mainly from tissue-resident stem cells rather than infiltrating immune or dedifferentiated mature cells (1). These progenitor cells differentiate into osteogenic and connective tissue lineages, reconstructing bone and surrounding tissues indistinguishable from the original structure.

While the role of blood vessels in digit tip regeneration has been documented (5, 6), the localization and function of lymphatic vessels in digit regeneration remain elusive. Beyond their traditional roles in fluid drainage, lipid absorption, and immune surveillance, lymphatic vessels are increasingly recognized for additional organ-specific functions (7, 8). For instance, lymphatic endothelial cells (LECs) can provide potent paracrine factors and facilitate immune responses across tissues.

In the current issue of the *JCI*, Vishlaghi et al. have demonstrated that lymphatic vessels surrounding the outer surface of the digit bone actively influence regeneration (9). They showed that lymphatics modulated local immune cell dynamics and that their ablation increased myeloid-to-osteoclast differentiation. The resulting rise in osteoclast number and activity enhanced osteolysis at the injury site, enabling quicker, more robust digit tip regeneration. These findings reveal that lymphatic vessels are not merely passive conduits but active regulators of bone remodeling and repair during digit tip regeneration.

## Experimental models inhibiting lymphangiogenesis improve digit tip regeneration

It is well established that lymphangiogenesis depends critically on signaling via the growth factor VEGF-C and its receptor, the transmembrane tyrosine kinase VEGFR3 (10). To investigate the link between lymphatics and bone regeneration outcome following P3 amputation, Vishlaghi et al. employed a combination of pharmacological and genetic models. Approaches involved the administration of a pharmacological VEGFR3 inhibitor, a genetic approach allowing the depletion of VEGFR3^+^ cells, or analysis of *Chy* mice, which exhibit constitutively diminished VEGFR3 activity following a heterozygous gene mutation and are considered a model of human lymphedema. In each model of impaired lymphangiogenesis, osteolysis of damaged bone was increased, which subsequently led to a higher bone mass in the regenerated digit (9). The uniformity of these results across different models is striking, yet the involvement of VEGFR3 also presents an important limitation: Experimental manipulations were not region specific, raising the possibility of systemic and therefore potentially indirect effects caused by alterations elsewhere in the lymphatic vasculature. In addition, VEGFR3 is required in certain blood vessels, such as the sinusoidal endothelial cells of the bone marrow (11, 12), which might lead to alterations in hematopoietic cell production in these models.

However, the authors also introduced a surgical model, in which the role of the lymphatics is restricted following lymph node removal (9). The added value of this surgical model lies in its independence from VEGFR3 manipulation, thereby ruling out potential off-target effects on sinusoidal endothelial cells from the bone marrow. Importantly, the lymph node removal followed by P3 digit tip amputation recapitulated the enhanced osteolysis and improved bone regeneration that Vishlaghi et al. identified following pharmacological and genetic suppression of VEGFR3, as described above. Together, these findings indicate that suppression of lymphangiogenesis indeed leads to improved digit tip regeneration.

## Potential mechanisms underlying enhanced digit regeneration

How might the targeting of lymphatic vessels promote early osteolysis to increase subsequent bone regeneration in the P3 amputation model? Following lymphatic inhibition, Vishlaghi et al. observed an increase in F4/80^+^ macrophages and enhanced myeloid-to-osteoclast differentiation (Figure 1C) (9). This is consistent with previous work showing that the depletion of F4/80^+^ macrophages impairs osteolysis, blastema formation, and tip regeneration (3). Vishlaghi et al. also reported that lymphatic vessel inhibition increased the abundance of T cells in the injured digit, which the authors identified as an important source of RANKL, a factor promoting osteoclastogenesis. It would be of interest to further characterize the T cell populations present at the injury site, given their capacity to regulate lymphatic vessel growth, but also osteoclast formation positively or negatively, depending on T cell subset and context (13, 14). Furthermore, macrophages have been shown to stimulate lymphangiogenesis by releasing VEGF-C and the related growth factor VEGF-D (15). In this model, lymphatic inhibition may trigger macrophage expansion by creating a feedback loop in which macrophages accumulate in an effort to restore LECs.

Previous work has indicated that ingrowth of lymphatic vessels into bone triggers osteolysis, as is the case in the rare human syndrome Gorham-Stout disease (GSD). In mouse models, GSD-like defects can be induced by excessive lymphangiogenesis, triggered by overexpression of VEGF-C, and might be linked to LEC-specific production of M-CSF, a protein promoting osteoclast formation and survival (16, 17). While the distinct and seemingly opposite roles of lymphatic vessels in animal models of GSD and in Vishlaghi et al. may appear puzzling, their location may help explain this discrepancy, as LECs remain confined to the surrounding tissue and do not penetrate the bone or blastema during digit regeneration. As lymphatic vessels facilitate immune cell trafficking, their ablation could disrupt an important route for immune cell exit, leading to the retention of immune cells, including the F4/80^+^ macrophages and T cells that Vishlaghi et al. linked with enhanced regeneration (9), at the injury site.

## Future outlook

While the exact mechanism linking impaired lymphatic vessel growth to improved digit tip regeneration deserves further investigation, the findings of this paper are valuable for advancing our understanding of digit tip regeneration and might have implications for bone fracture healing. Although tip regeneration and bone fracture healing are distinct repair processes, they share notable parallels, such as the essential role of osteoclasts, which are required for hard callus remodeling and for the resorption of necrotic bone fragments preceding new bone formation (18). While lymphatic vessels surround bone throughout the skeletal system (19), the reorganization of this network and its function during regeneration and fracture healing have remained elusive. In addition, elucidating the contributions of LEC-derived paracrine (lymphangiocrine) signals (7), of lymphatic drainage, and of alterations in the trafficking of immune cells would provide important insight into lymphatic-immune crosstalk in the observations of Vishlaghi and colleagues.

It would also be of interest to test whether lymphatic ablation could confer regenerative capacity to the typically non-regenerative P2-level amputation. Previous studies have shown that the application of growth factors or transplantation of pluripotent stem cells can promote P2 digit regeneration (20, 21). Conversely, experiments in which enhanced lymphangiogenesis impairs P3 digit regeneration would further strengthen the proposed mechanistic concept.

From a therapeutic perspective, surgical removal of lymph nodes or systemic inhibition of lymphangiogenesis would be problematic due to the associated risks of lymphedema and increased inflammation (22, 23). However, it might be feasible to transiently modulate lymphatic signaling pathways to facilitate bone formation.

Together, it will be very interesting to see how future research continues to explore the multifaceted role of lymphatic vessels in regenerative processes.

## Funding support

- ML by the Rubicon grant funded by ZonMw (04520232410013).
- FLB and RHA by the MPI for Molecular Biomedicine.
- FLB and RHA by the Max Planck Society.

## Version 1 02/02/2026

Electronic publication
